# Supplementary material for: SARS-CoV-2 viral persistence in lung alveolar macrophages is controlled by IFN-γ and NK cells
Source: Nat Immunol. 2023 Nov 2;24(12):2068–79. doi: 10.1038/s41590-023-01661-4 (PMC10681903; doi:10.1038/s41590-023-01661-4)
Supplement: Supplementary file 1 — Supplementary Table 1: list of the antibodies used for flow cytometry and immunofluorescence staining. [file 41590_2023_1661_MOESM1_ESM.pdf]

nature immunology

Article

https://doi.org/10.1038/s41590-023-01661-4

SARS-CoV-2 viral persistence in lung alveolar macrophages is controlled by IFN-γ and NK cells

In the format provided by the authors and unedited

Antibody used for flow analysed

Extra¢	Fluorochrome	Providers	Clone	Isotype	Reference	Control	NHP Cross reactivity	Dilution
NKG2A (CD159a)	PE	Coulter	Z199	IgG2b	IM3291U	Isotype + FMO	Providers + NHP reagent	1/30
CD3ε	ECD/PE-C594	BD Biosciences	SP34,2	IgG1, λ	562406	Isotype + FMO	Providers + NHP reagent	1/15
CD16	BUV496	BD Biosciences	3G8	612944	IgG1, κ	Isotype + FMO	Providers + NHP reagent	1/20
CD45	BUV395	BD Biosciences	D058-1283	564099	IgG1, κ	Isotype + FMO	Providers + NHP reagent	1/30
CD4	PerCp-Cy5	BD Biosciences	L200	IgG1, κ	552838	Isotype + FMO	Providers + NHP reagent	1/15
CD336 (NKp44)	PC7	Miltenyi Biotec	REA1163	IgG1	130-120-359	Isotype + FMO	Providers + NHP reagent	1/20
CD337 (NKp30)	APC	Miltenyi Biotec	REA823	IgG1	130-112-431	Isotype + FMO	Providers + NHP reagent	1/20
CD107a	A700	BD Biosciences	H4A3	IgG1, κ	561340	Isotype + FMO	Providers + NHP reagent	1/10
NKp80	APC Vio® 770	Miltenyi Biotec	REA845	IgG1	130-112-593	Isotype + FMO	Providers + NHP reagent	1/20
CD226	BV605	BD Biosciences	DX11	IgG1, κ	742495	Isotype + FMO	Providers + NHP reagent	1/15
CD20	BV711	BD Biosciences	2H7	IgG2b, κ	563126	Isotype + FMO	Providers + NHP reagent	1/30
CD103	BV650	BD Biosciences	Ber-ACT8	IgG1, κ	743653	Isotype + FMO	Providers + NHP reagent	1/20
KI-67	AF®488	BD Biosciences	B56	IgG1, κ	561165	Isotype + FMO	Providers + NHP reagent	1/20
GZMB	V450	BD Biosciences	GB11	561151	178,75	Isotype + FMO	Providers + NHP reagent	1/15
IFN-γ	BV510	BD Biosciences	B27	563287	159,25	Isotype + FMO	Providers + NHP reagent	1/20
IL-17	BV786	BioLegend	BL168	512337	127,92	Isotype + FMO	Providers + NHP reagent	1/20
CD4	PE-Cy™7	BD Biosciences	L200	IgG1, κ	560644	Isotype + FMO	Providers + NHP reagent	1/20
CD8	VioBlue®	Miltenyi Biotec	Bw135/80	IgG2aκ	130-113-162	Isotype + FMO	Providers + NHP reagent	1/30
CD7	BV650	BD Biosciences	M-T701	IgG1, κ	740565	Isotype + FMO	Providers + NHP reagent	1/15
HLA-DR	AF®700	BD Biosciences	L243 (G46-6)	IgG2a, κ	560743	Isotype + FMO	Providers + NHP reagent	1/20
CD20	BV711	BD Biosciences	2H7	IgG2b, κ	563126	Isotype + FMO	Providers + NHP reagent	1/20
CD45RA	BV786	BD Biosciences	5H9	IgG1, κ	741010	Isotype + FMO	Providers + NHP reagent	1/20
CD34	BUV737	BD Biosciences	563	IgG1, κ	741868	Isotype + FMO	Providers + NHP reagent	1/15
EOMES	FITC	Invitrogen	WD1928	IgG1, κ	11-4877-42	Isotype + FMO	Providers + NHP reagent	1/30
T-bet	PerCP/Cy5.5	SONY	4B10	IgG1, κ	RT3824030	Isotype + FMO	Providers + NHP reagent	1/10
IL-21	AF®647	BD Biosciences	3A3-N2.1	IgG1	562043	Isotype + FMO	Providers + NHP reagent	1/40
IFN-γ	APC/Cy7	SONY	B27	IgG1, κ	RT3132620	Isotype + FMO	Providers + NHP reagent	1/15
CD20	A700	BD Biosciences	2H7	IgG2b, κ	560631	Isotype + FMO	Providers + NHP reagent	1/30
CD34	BUV737	BD Biosciences	563	IgG1, κ	741868	Isotype + FMO	Providers + NHP reagent	1/20
CD45RA	BV786	BD Biosciences	5H9	IgG1, κ	741010	Isotype + FMO	Providers + NHP reagent	1/20
CD7	BV650	BD Biosciences	M-T701	IgG1, κ	740565	Isotype + FMO	Providers + NHP reagent	1/20
CCR7	APC/Cy7	BioLegend	G043H7	IgG2a, κ	353211	Isotype + FMO	Providers + NHP reagent	1/15
CXCR3 (CD183)	PE-Cy™7	BD Biosciences	1C6	IgG1, κ	560831	Isotype + FMO	Providers + NHP reagent	1/30
CD44	BUV661	BD Biosciences	G44-26	IgG2b, κ	741615	Isotype + FMO	Providers + NHP reagent	1/20
CD127	PE-Cy5	Invitrogen	eBioRDR5	IgG1 κ	15-1278-42	Isotype + FMO	Providers + NHP reagent	1/20
GATA-3	BV421/450	BD Biosciences	L50-823	IgG1, κ	563349	Isotype + FMO	Providers + NHP reagent	1/20
T-bet	PerCP/Cy5.5	SONY	4B10	IgG1, κ	RT3824030	Isotype + FMO	Providers + NHP reagent	1/20
CD14	alexa 488, FITC,BB515	Miltenyi Biotec	TÜK4	IgG2aκ	130-113-146	Isotype + FMO	Providers + NHP reagent	1/10
MHC-E	PE	NOVUS	3D12MHLA-E	IgG1, κ	NBP2-00277	Isotype + FMO	Providers + NHP reagent	1/30
CD206	ECD	BD Biosciences	19.2	IgG1,	564063	Isotype + FMO	Providers + NHP reagent	1/20
IL-10	PC7	BioLegend	JES3-9D7	IgG1, κ	501419	Isotype + FMO	Providers + NHP reagent	1/20
CD64	APC	BD Biosciences	10,1	IgG2b, κ	561189	Isotype + FMO	Providers + NHP reagent	1/15
CD11C	BV605	BD Biosciences	S-HCL-3	IgG2b, κ	744436	Isotype + FMO	Providers + NHP reagent	1/20
NKG2c	Vio® Bright R720	Miltenyi Biotec	REA205	IgG2b, κ	130-130-663	Isotype + FMO	Providers + NHP reagent	1/10
CD163	BV421/450	BioLegend	GHI/61	IgG1,	333619	Isotype + FMO	Providers + NHP reagent	1/20

Antibody used for Immunostainig

Target	Primary antibody	Secondary antibody	Providers	Clone	Reference	Dilution
NSP3	Yes	No	abcam	na	ab283958	1/300
dsRNA	Yes	No	Millipore	rJ2	MABE1134	1/250
SPIKE	Yes	No	see method	Cv2.3194	Reference 49	1/500
ACTIN	No	No	ThermoFisher	na	A12379	providers
MHC-E	Yes	No	Invitrogen	MEM-E/0(	MA1-19356	1/250
IL-10	Yes	No	BD bioscience	JES3-19F1	554704	1/150
IFNg	Yes	No	eBioscience	MD-1	16-7317-81	1/200
IL-18	Yes	No	ATLAS antibody	na	HPA003980	1/200
IL-1b	Yes	No	abcam	na	ab2105	1/200
IL-23	Yes	No	abcam	B-Z23	ab84471	1/300
