# Supplementary material for: SARS-CoV-2 viral persistence in lung alveolar macrophages is controlled by IFN-γ and NK cells
Source: Nat Immunol. 2023 Nov 2;24(12):2068–79. doi: 10.1038/s41590-023-01661-4 (PMC10681903; doi:10.1038/s41590-023-01661-4)
Supplement: Supplementary file 2 — Reporting Summary [file 41590_2023_1661_MOESM2_ESM.pdf]

Corresponding author(s): Huot Nicolas

Last updated by author(s): Sep 20, 2023

Reporting Summary

Nature Portfolio wishes to improve the reproducibility of the work that we publish. This form provides structure for consistency and transparency in reporting. For further information on Nature Portfolio policies, see our Editorial Policies and the Editorial Policy Checklist.

Statistics

For all statistical analyses, confirm that the following items are present in the figure legend, table legend, main text, or Methods section.

n/a	Confirmed	
☐	☒	The exact sample size (n) for each experimental group/condition, given as a discrete number and unit of measurement
☐	☒	A statement on whether measurements were taken from distinct samples or whether the same sample was measured repeatedly
☐	☒	The statistical test(s) used AND whether they are one- or two-sided<br>*Only common tests should be described solely by name; describe more complex techniques in the Methods section.*
☐	☒	A description of all covariates tested
☐	☒	A description of any assumptions or corrections, such as tests of normality and adjustment for multiple comparisons
☐	☒	A full description of the statistical parameters including central tendency (e.g. means) or other basic estimates (e.g. regression coefficient) AND variation (e.g. standard deviation) or associated estimates of uncertainty (e.g. confidence intervals)
☐	☒	For null hypothesis testing, the test statistic (e.g. F, t, r) with confidence intervals, effect sizes, degrees of freedom and P value noted<br>*Give P values as exact values whenever suitable.*
☒	☐	For Bayesian analysis, information on the choice of priors and Markov chain Monte Carlo settings
☒	☐	For hierarchical and complex designs, identification of the appropriate level for tests and full reporting of outcomes
☐	☒	Estimates of effect sizes (e.g. Cohen's d, Pearson's r), indicating how they were calculated

Our web collection on statistics for biologists contains articles on many of the points above.

Software and code

Policy information about availability of computer code

Data collection	Flow cytometry analysis was performed using a Fortessa (BD Biosciences) equipped with DIVA software (version 8.0). Immunofluorescence analysis was carried out using either a CELENA® X High Content Imaging System (LOGOS BIOSYSTEMS, South Korea) with the CELENA® X Explorer (version 1.6.0) or a spinning-disk Ti2E Microscope (Nikon - Yokagawa CSU W1). Thermal cycling was conducted in a 7500 real-time PCR system (Applied Biosystems) equipped with Windows 10 and 7500 Fast software version 2.3.
Data analysis	The experimental group size estimation was performed using G Power software version 3.1.9.7, and fluorescence quantification and cell segmentation were carried out using the Fiji software (version 1.53 with Java 1.8.0_322). In the quest to identify SARS-CoV-2 peptides with the potential for efficient binding to MHC-E, IEBD prediction tools (version 2.27) were employed, and Signalp software (version 6.0) was used for further analysis. Cytometry analysis was conducted with FlowJo software (version 10.4.2, Tree Star, Ashland, OR). Subsequently, data analysis and statistical tests were performed using GraphPad Prism 7 (GraphPad Software, version 10.0.2, San Diego, CA).

For manuscripts utilizing custom algorithms or software that are central to the research but not yet described in published literature, software must be made available to editors and reviewers. We strongly encourage code deposition in a community repository (e.g. GitHub). See the Nature Portfolio guidelines for submitting code & software for further information.

Data

Policy information about availability of data

All manuscripts must include a data availability statement. This statement should provide the following information, where applicable:

- Accession codes, unique identifiers, or web links for publicly available datasets
- A description of any restrictions on data availability
- For clinical datasets or third party data, please ensure that the statement adheres to our policy

All the data that support the study's findings are included either within the article itself or are provided in a format that readers can access directly from the article. The authors provide raw data files associated with the study. These files likely contain detailed data points and information related to the research. all figures presented in the manuscript are linked to the corresponding raw data files.
All omics data are deposited in public repositories and access codes specified in the paper.

Field-specific reporting

Please select the one below that is the best fit for your research. If you are not sure, read the appropriate sections before making your selection.

☒ Life sciences ☐ Behavioural & social sciences ☐ Ecological, evolutionary & environmental sciences

For a reference copy of the document with all sections, see nature.com/documents/nr-reporting-summary-flat.pdf

Life sciences study design

All studies must disclose on these points even when the disclosure is negative.

Sample size	We followed the "3Rs" principle for animal research, aiming to minimize the use of experimental subjects. Animals were selected to closely match in terms of age, weight, and genotype. Sample size calculations were performed using power analysis, which accounts for effect size, standard deviation, type 1 error, and 80% power in a two-sample t-test with a 5% significance level (two-sided test). We used G power software version 3.1.9.7. for these calculations, with D determined as (difference in means)/(standard deviation), estimated from our preliminary results. We considered a maximum D value of 1.8 based on previous studies for sample size calculation.
Data exclusions	No data were excluded from the analyses.
Replication	Some assays were performed as replicates: viral quantifications and competitive analysis for binding of the peptides to MHC. The latter comprised three replicates (three independent experiments for each condition). We did not observe any major variation with all the replicates. All results were included in the manuscript. For the other assays, we only used biological replica (ie distinct animals). No other attempts were made at replication due to the limitation in volume of samples, and also duration of the in vivo study (2 years), substantial costs associated with animal acquisition, per diem charges, and surgical costs.
Randomization	Sample collection and analyses were performed in random order.
Blinding	The investigators were not blinded while the animal handlers were blinded to group allocation.

Reporting for specific materials, systems and methods

We require information from authors about some types of materials, experimental systems and methods used in many studies. Here, indicate whether each material, system or method listed is relevant to your study. If you are not sure if a list item applies to your research, read the appropriate section before selecting a response.

Materials & experimental systems

n/a	Involved in the study	
☐	☒	Antibodies
☐	☒	Eukaryotic cell lines
☒	☐	Palaeontology and archaeology
☐	☒	Animals and other organisms
☐	☒	Human research participants
☒	☐	Clinical data
☒	☐	Dual use research of concern

Methods

n/a	Involved in the study	
☒	☐	ChIP-seq
☐	☒	Flow cytometry
☒	☐	MRI-based neuroimaging

Antibodies

Antibodies used	These antibodies, along with their respective characteristics, were crucial for ensuring the accuracy and reliability of our research findings.

CD45, labeled with BUV395 and provided by BD Biosciences (Clone: D058-1283, Isotype: IgG1, κ, Reference: 564099), was used at a dilution of 1/30. Control conditions included Isotype + FMO and Providers + NHP reagent. CD4, labeled with PerCp-Cy5 and sourced from BD Biosciences (Clone: L200, Isotype: IgG1, κ, Reference: 552838), was utilized at a dilution of 1/15. Control conditions comprised Isotype + FMO and Providers + NHP reagent.

CD336 (NKp44), labeled with PC7 and obtained from Miltenyi Biotec (Clone: REA1163, Isotype: IgG1, Reference: 130-120-359), was used at a dilution of 1/20. Control conditions included Isotype + FMO and Providers + NHP reagent.CD337 (NKp30), labeled with APC and sourced from Miltenyi Biotec (Clone: REA823, Isotype: IgG1, Reference: 130-112-431), was employed at a dilution of 1/20. Control conditions comprised Isotype + FMO and Providers + NHP reagent.CD107a, labeled with A700 and provided by BD Biosciences (Clone: H4A3, Isotype: IgG1, κ, Reference: 561340), was used at a dilution of 1/10. Control conditions included Isotype + FMO and Providers + NHP reagent. NKp80, labeled with APC Vio® 770 and sourced from Miltenyi Biotec (Clone: REA845, Isotype: IgG1, Reference: 130-112-593), was utilized at a dilution of 1/20. Control conditions comprised Isotype + FMO and Providers + NHP reagent. CD226, labeled with BV605 and obtained from BD Biosciences (Clone: DX11, Isotype: IgG1, κ, Reference: 742495), was used at a dilution of 1/15. Control conditions included Isotype + FMO and Providers + NHP reagent. CD20, labeled with BV711 and provided by BD Biosciences (Clone: 2H7, Isotype: IgG2b, κ, Reference: 563126), was employed at a dilution of 1/30. Control conditions comprised Isotype + FMO and Providers + NHP reagent. CD103, labeled with BV650 and sourced from BD Biosciences (Clone: Ber-ACT8, Isotype: IgG1, κ, Reference: 743653), was used at a dilution of 1/20. Control conditions included Isotype + FMO and Providers + NHP reagent. KI-67, labeled with AF®488 and obtained from BD Biosciences (Clone: B56, Isotype: IgG1, κ, Reference: 561165), was utilized at a dilution of 1/20. Control conditions comprised Isotype + FMO and Providers + NHP reagent. GZMB, labeled with V450 and provided by BD Biosciences (Clone: GB11, Isotype: IgG1, Reference: 561151), was used at a dilution of 1/15. Control conditions included Isotype + FMO and Providers + NHP reagent. IFN-γ, labeled with BV510 and sourced from BD Biosciences (Clone: B27, Isotype: IgG1, κ, Reference: 563287), was employed at a dilution of 1/20. Control conditions comprised Isotype + FMO and Providers + NHP reagent. IL-17, labeled with BV786 and obtained from BioLegend (Clone: BL168, Isotype: IgG1, Reference: 512337), was utilized at a dilution of 1/20. Control conditions included Isotype + FMO and Providers + NHP reagent. CD4, labeled with PE-Cy™7 and sourced from BD Biosciences (Clone: L200, Isotype: IgG1, κ, Reference: 560644), was used at a dilution of 1/20. Control conditions comprised Isotype + FMO and Providers + NHP reagent. CD8, labeled with VioBlue® and obtained from Miltenyi Biotec (Clone: Bw135/80, Isotype: IgG2aκ, Reference: 130-113-162), was employed at a dilution of 1/30. Control conditions included Isotype + FMO and Providers + NHP reagent. CD7, labeled with BV650 and provided by BD Biosciences (Clone: M-T701, Isotype: IgG1, κ, Reference: 740565), was used at a dilution of 1/15. Control conditions comprised Isotype + FMO and Providers + NHP reagent. HLA-DR, labeled with AF®700 and sourced from BD Biosciences (Clone: L243 (G46-6), Isotype: IgG2a, κ, Reference: 560743), was utilized at a dilution of 1/20. Control conditions included Isotype + FMO and Providers + NHP reagent. CD20, labeled with BV711 and obtained from BD Biosciences (Clone: 2H7, Isotype: IgG2b, κ, Reference: 563126), was employed at a dilution of 1/20. Control conditions comprised Isotype + FMO and Providers + NHP reagent. CD45RA, labeled with BV786 and sourced from BD Biosciences (Clone: 5H9, Isotype: IgG1, κ, Reference: 741010), was used at a dilution of 1/20. Control conditions included Isotype + FMO and Providers + NHP reagent. CD34, labeled with BUV737 and provided by BD Biosciences (Clone: 563, Isotype: IgG1, κ, Reference: 741868), was utilized at a dilution of 1/15. Control conditions comprised Isotype + FMO and Providers + NHP reagent. EOMES, labeled with FITC and sourced from Invitrogen (Clone: WD1928, Isotype: IgG1, κ, Reference: 11-4877-42), was used at a dilution of 1/30. Control conditions included Isotype + FMO and Providers + NHP reagent. T-bet, labeled with PerCP/Cy5.5 and obtained from SONY (Clone: 4B10, Isotype: IgG1, κ, Reference: RT3824030), was employed at a dilution of 1/10. Control conditions comprised Isotype + FMO and Providers + NHP reagent. IL-21, labeled with AF®647 and provided by BD Biosciences (Clone: 3A3-N2.1, Isotype: IgG1, Reference: 562043), was used at a dilution of 1/40. Control conditions included Isotype + FMO and Providers + NHP reagent. IFN-γ, labeled with APC/Cy7 and sourced from SONY (Clone: B27, Isotype: IgG1, κ, Reference: RT3132620), was employed at a dilution of 1/15. Control conditions comprised Isotype + FMO and Providers + NHP reagent. CD20, labeled with A700 and obtained from BD Biosciences (Clone: 2H7, Isotype: IgG2b, κ, Reference: 560631), was used at a dilution of 1/30. Control conditions included Isotype + FMO and Providers + NHP reagent. CD34, labeled with BUV737 and provided by BD Biosciences (Clone: 563, Isotype: IgG1, κ, Reference: 741868), was utilized at a dilution of 1/20. Control conditions comprised Isotype + FMO and Providers + NHP reagent. CD45RA, labeled with BV786 and sourced from BD Biosciences (Clone: 5H9, Isotype: IgG1, κ, Reference: 741010), was used at a dilution of 1/20. Control conditions included Isotype + FMO and Providers + NHP reagent. CCR7, labeled with APC/Cy7 and sourced from BioLegend (Clone: G043H7, Isotype: IgG2a, κ, Reference: 353211), was utilized at a dilution of 1/15. Control conditions included Isotype + FMO and Providers + NHP reagent. CXCR3 (CD183), labeled with PE-Cy™7 and obtained from BD Biosciences (Clone: 1C6, Isotype: IgG1, κ, Reference: 560831), was employed at a dilution of 1/30. Control conditions comprised Isotype + FMO and Providers + NHP reagent. CD44, labeled with BUV661 and provided by BD Biosciences (Clone: G44-26, Isotype: IgG2b, κ, Reference: 741615), was used at a dilution of 1/20. Control conditions included Isotype + FMO and Providers + NHP reagent. CD127, labeled with PE-Cy5 and sourced from Invitrogen (Clone: eBioRDR5, Isotype: IgG1 κ, Reference: 15-1278-42), was employed at a dilution of 1/20. Control conditions comprised Isotype + FMO and Providers + NHP reagent. GATA-3, labeled with BV421/450 and obtained from BD Biosciences (Clone: L50-823, Isotype: IgG1, κ, Reference: 563349), was utilized at a dilution of 1/20. Control conditions included Isotype + FMO and Providers + NHP reagent.

T-bet, labeled with PerCP/Cy5.5 and sourced from SONY (Clone: 4B10, Isotype: IgG1, κ, Reference: RT3824030), was used at a dilution of 1/20. Control conditions comprised Isotype + FMO and Providers + NHP reagent. CD14, labeled with alexa 488, FITC, and BB515, was obtained from Miltenyi Biotec (Clone: TÜK4, Isotype: IgG2aκ, Reference: 130-113-146). It was used at a dilution of 1/10, and control conditions included Isotype + FMO and Providers + NHP reagent. MHC-E, labeled with PE and provided by NOVUS (Clone: 3D12MHLA-E, Isotype: IgG1, κ, Reference: NBP2-00277), was used at a dilution of 1/30. Control conditions comprised Isotype + FMO and Providers + NHP reagent. CD206, labeled with ECD and sourced from BD Biosciences (Clone: 19.2, Isotype: IgG1), was employed in our analysis. Control conditions included Isotype + FMO and Providers + NHP reagent. IL-10, labeled with PC7 and obtained from BioLegend (Clone: JES3-9D7, Isotype: IgG1, κ, Reference: 501419), was utilized in our experiments. Control conditions comprised Isotype + FMO and Providers + NHP reagent. CD64, labeled with APC and provided by BD Biosciences (Clone: 10,1, Isotype: IgG2b, κ, Reference: 561189), was used in our analysis. Control conditions included Isotype + FMO and Providers + NHP reagent. CD11C, labeled with BV605 and sourced from BD Biosciences (Clone: S-HCL-3, Isotype: IgG2b, κ, Reference: 744436), was employed in our experiments. Control conditions comprised Isotype + FMO and Providers + NHP reagent. NKG2c, labeled with Vio® Bright R720 and obtained from Miltenyi Biotec (Clone: REA205, Isotype: IgG2b, κ, Reference: 130-130-663), was utilized in our analysis. Control conditions included Isotype + FMO and Providers + NHP reagent. CD163, labeled with BV421/450 and provided by BioLegend (Clone: GHI/61, Isotype: IgG1), was used in our experiments. Control conditions comprised Isotype + FMO and Providers + NHP reagent.

Validation

the antibodies employed for flow cytometry analysis underwent a thorough validation process to ensure their reliability and accuracy in detecting specific immune cell populations and markers. The validation process included several critical steps:

Antibody Selection: The initial step in the validation process involved the careful selection of antibodies. Antibodies were chosen

based on their known specificity for the target antigens of interest. Extensive literature review and consultation with experts in the field were conducted to confirm their suitability for our study.

Manufacturer's Specifications: We reviewed the manufacturer's specifications and technical data sheets for each antibody. This information included details such as clone names, isotypes, recommended dilutions, and references. Understanding these specifications was essential for proper antibody usage.

Positive and Negative Controls: To validate antibody performance, we used positive and negative controls. Positive controls included cells or tissues known to express the target antigen, while negative controls were cells or tissues lacking the antigen. These controls helped confirm the specificity of the antibodies.

Fluorescence Minus One (FMO) Controls: FMO controls were utilized to assess background fluorescence and determine the true signal from the target antigen. FMO controls included all fluorochromes except the one specific to the tested antibody, allowing us to differentiate specific staining from non-specific background fluorescence.

Non-Human Primate (NHP) Reagent Controls: Since our study involved non-human primates, NHP reagent controls were used to ensure the compatibility and specificity of the antibodies in the primate model. These controls helped verify that the antibodies effectively bound to the target antigens in our experimental system.

Dilution Optimization: Antibody dilutions were optimized to achieve the best signal-to-noise ratio. Serial dilutions of each antibody were tested to determine the optimal concentration for accurate and robust staining.

Eukaryotic cell lines

Policy information about cell lines

Cell line source(s)	K562 cell line was provided by the ATCC ; The K-562 cell line was purchased from ATCC and transduced with a lentivirus expression vector for human HLA-E was purchases from Applied Biological Materials Inc.
Authentication	None of the cell lines were authenticated.
Mycoplasma contamination	We confirm that the cell line was tested negative for mycoplasma
Commonly misidentified lines (See ICLAC register)	ATCC® Number: CCL-243™ ; no commonly misidentified cell lines were used in the study.

Animals and other organisms

Policy information about studies involving animals; ARRIVE guidelines recommended for reporting animal research

Laboratory animals	Cynomolgus macaques (M. fascicularis) aged 37–60 months were sourced from Mauritian AAALAC-certified breeding centers. The study was conducted at IDMIT infrastructure facilities with appropriate containment levels in accordance with regulations. At the time of infection, all animals were seronegative for specific viruses. Following infection, the macaques were housed individually in biosafety facilities. Both male and female monkeys were included in the study, and sample collection was performed randomly
Wild animals	Study did not involve wild animals.
Field-collected samples	Study did not involve field-collected samples
Ethics oversight	The study protocols received approval from the institutional ethical committee, 'Comité d'Ethique en Expérimentation Animale du Commissariat à l'Energie Atomique et aux Energies Alternatives' (CEtEA 44), under statement numbers A20_011, 20_066, and 21_069. Authorization for the study was granted by the 'Research, Innovation, and Education Ministry' under registration numbers APAFIS#24434–2020030216532863v1, APAFIS#29191-2021011811505374 v1, and APAFIS#33414-2021101115102064 v1.

Note that full information on the approval of the study protocol must also be provided in the manuscript.

Human research participants

Policy information about studies involving human research participants

Population characteristics	Blood samples was collected based on voluntary. To be allowed to give blood sample, Volunteers should had between 18 and 70 years old, and weigh more than 50 kg. Exact age, sexe and ethnic origin of the participantes is not known. Fifty milliliter blood samples were obtained from twelve donors.
Recruitment	Blood samples was collected based on voluntary. All blood samples were collected before 2019 and PBMC were isolated using Ficoll gradient and immediately frozen.
Ethics oversight	Blood samples from healthy donors were obtained from the French blood bank (Etablissement Français du Sang) as part of an agreement with the Institut Pasteur (C CPSL UNT, number 15/EFS/023). The study was approved by the Ethics Review Committee (Comité de protection des personnes) of Île-de-France VII.

Note that full information on the approval of the study protocol must also be provided in the manuscript.

Flow Cytometry

Plots

Confirm that:

☒ The axis labels state the marker and fluorochrome used (e.g. CD4-FITC).

☒ The axis scales are clearly visible. Include numbers along axes only for bottom left plot of group (a 'group' is an analysis of identical markers).

☒ All plots are contour plots with outliers or pseudocolor plots.

☒ A numerical value for number of cells or percentage (with statistics) is provided.

Methodology

Sample preparation	Whole venous blood was collected in ethylenediaminetetraacetic acid (EDTA) tubes. Peripheral blood mononuclear cells (PBMCs) were isolated by Ficoll density-gradient centrifugation. Broncho alveolar lavage fluid have been collected when bllod puncture was done. The cell suspension was subsequently filtered through 100- and 40-µm cell strainers, and cells were washed with cold phosphate-buffered saline (PBS). Cells were either immediately stained for flow cytometry or cryopreserved in 90% foetal bovine serum (FBS) and 10% dimethyl sulfphoxide (DMSO) and stored in liquid nitrogen before use. Intracellular staining was performed using BD Cytofix/Cytoperm™.
Instrument	Flow cytometry acquisitions were done on a LSRFortessa (BD Biosciences).
Software	The data were analysed using Diva (software version 6.1.3, build 2009 05 13 13 29). The data were further analyzed using FlowJo (10.4.2 software, LLC, Ashland, OR, USA). Multiparametric analyses were performed using SPICE (version 5.1). t-SNE was performed with the cytobank (Cytobank, Inc.), using 2,000 iterations and a perplexity of 60.
Cell population abundance	Online methods, paragraph: Polychromatic flow cytometry ; Cell sorting of NK cell sub-populations.
Gating strategy	all gating strategies are provided in extended figures

☒ Tick this box to confirm that a figure exemplifying the gating strategy is provided in the Supplementary Information.
